# Phthiriasis capitis *ab initio*. Use of entodermoscopy for quick differentiation between *Phthirus pubis* and *Pediculus capitis* nits^⋆^^⋆⋆^

**DOI:** 10.1016/j.abd.2020.03.019

**Published:** 2020-09-16

**Authors:** Gaetano Scanni

**Affiliations:** Service of Medicine, Azienda Sanitaria Locale Della Província di Bari, Bari, Italy

Dear Editor,

*Phthiriasis pubis* (*crab louse infestation*) is an ectoparasitosis transmitted mostly by skin to skin contact, primarily located in the genital areas and secondly, if treated late, expanding towards the armpits or other parts of the adult body with thick hair. In children before sexual maturation, the affected sites can include the eyelashes or head hair, usually after contact with an adult who has been sexually infected. Commonly, diagnosis is easy and requires finding eggs (nits) and/or insects, generally firmly hooked to hair shaft base. Because phthiriasis is included in sexually transmitted diseases, it is recommended to expand investigation to other venereal diseases.

Recent reports about *Phthirus pubis* seem to suggest a change of its usual behavior, favoring a primary scalp location without genital involvement, as already described by some authors and observed in a personal unpublished series.^1^, ^2^, ^3^

Although fomites can carry parasites anywhere, cases considered in this letter refer to adults or school age children whose infestation was neither transmitted through objects or sexual intercourse, nor by non-sexual touching (i.e., when children sleep with a parent). A careful anamnestic reconstruction was only suggestive for a head-to-head direct contact modality.^4^

This scenario, not yet fully investigated, could be partially explained with some hypotheses. One of the most probable could be related to the very popular practice of pubis and underarm trichotomy or laser epilation of any part of the body that eliminates the parasite’s natural habitat. Another suggestive hypothesis could regard some connection between terrestrial climatic changes and different receptivity of human skin to *P. pubis*. Anyway, in such unusual conditions, crabs appear to skip genital targets and going directly towards cephalic areas.

If crab lice develop in specific context such as schools, where physical contacts are very common among children, there is a risk of misdiagnosis because school is the place where *Pediculus capitis* is normally expected as main cause of morbidity and head itch. Unfortunately, scalp itch and nits alone are not sufficient to formulate a correct differentiation with naked eye between *P. capitis* and *P. pubis*; on the contrary, it possible by entodermoscopy, which is dermatoscopy with an entomological focus. In fact, the nits of these two different species of parasite, although similar at first glance, have two pathognomonic parts that help to distinguish them: the operculum and the fixing sleeve to the hair shaft.^5^ *P. capitis* nits are closed on top by a dome drilled cover (operculum + aeropyles) and glued to hair shaft by a long, thin, tubular sleeve (Fig. 1). *P. pubis* nits instead have a conically shaped operculum and a short, thick sleeve (Fig. 2). These morphological features can be seen through a 10× magnification hand-held dermatoscope or much better with a 30–50× digital/video dermatoscope. While operculum is relevant for diagnosis in unhatched nits (Figure 1, Figure 2A), the fixing sleeve becomes more important to recognize nits when they lose the operculum because the nymph has hatched (Figure 1, Figure 2B).

**Figure 1:**
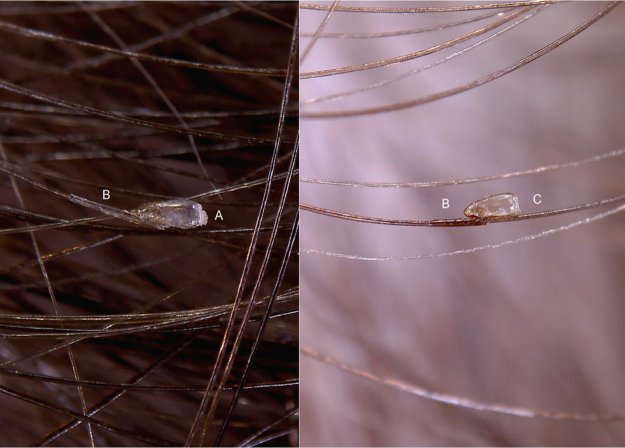
Entodermoscopy of pediculus capitis nit on scalp without liquid interface (×30). (A), dome-shaped operculum; (B), long and tubular fixing sleeve; (C), nit without operculum.

**Figure 2:**
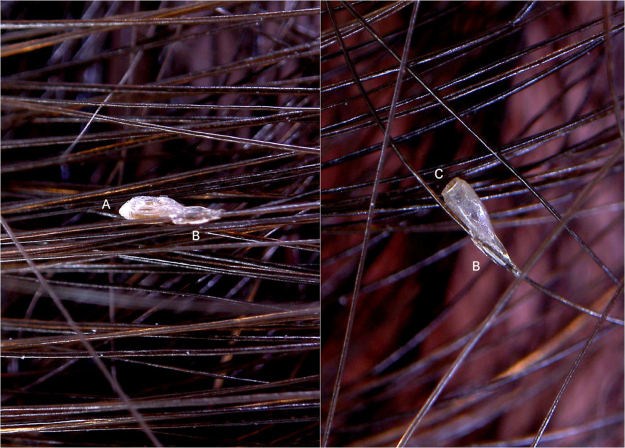
Entodermoscopy of *Phthirus pubis* nit on scalp without liquid interface (×30). (A), conically shaped operculum; (B), short and thick sleeve fixing to hair shaft; (C), nit without operculum.

In the future, it may be observed an increase in the diagnostic of primitive scalp infestations by *P. pubis*, until now underestimated, because symptoms and macroscopic affinity with the more common *P. capitis*. What makes cases considered in this letter interesting is that *P. pubis* can exhibit, when needed, primary interest not only for the usual biological niche of *P. capitis*, but also for the same modality of transmission (head-to-head).

On the occasion of scalp pruritus either in children or adults, the author recommends an extemporaneous dermoscopic examination of nits to differentiate what kind of pediculosis is really involved. This procedure is able to return the same anatomical structures observable by an optical microscope, but in an easier and faster way. Regarding therapy, it follows the currently guidelines for *P. capitis*, although the management of contacts can be more complex because of the possibility of sexual abuse in children, which must be ruled out.

In conclusion, when phthiriasis is diagnosed only on the head without any other site involved and without any type of sexual or non-sexual physical contact, the pathogen of this *ab initio* condition could be dubbed with the more appropriate name of *Phthirus capitis* rather *pubis*, as already proposed by some authors.^1^, ^2^ Lastly, although a mere opinion of the author, this original pediculus behavior could represent matter of speculation for entomologists and dermatologists because the possibility that a human obliged pathogen changes due to an evolutionary pressure by society’s habits and customs, and perhaps also by the impact of climate changes. Thus, entodermoscopy shows still to be a suitable tool for diagnosing an ectoparasitosis even when it is located on unexpected body sites.

## Financial support

None declared.

## Authors' contributions

Gaetano Scanni: Approval of the final version of the manuscript; design and planning of the study; drafting and editing of the manuscript; collection, analysis, and interpretation of data; effective participation in research orientation; intellectual participation in the propaedeutic and/or therapeutic conduct of the studied cases; critical review of the literature; critical review of the manuscript.

## Conflicts of interest

None declared.
